# Yield and nutrient composition of forage crops and their effects on soil characteristics of winter fallow paddy in South China

**DOI:** 10.3389/fpls.2023.1292114

**Published:** 2024-01-16

**Authors:** Liuxing Xu, Guojian Tang, Dan Wu, Jianguo Zhang

**Affiliations:** ^1^ College of Agronomy and Life Sciences, Zhaotong University, Zhaotong, China; ^2^ Department of Grassland Science, South China Agricultural University, Guangzhou, China; ^3^ School of Biological Sciences and Technology, Liupanshui Normal University, Liupanshui, China

**Keywords:** ecological benefit, nutritive composition, soil, yield, green manure

## Abstract

In terms of providing additional feeds and improving the soil fertility, planting forage crops during the fallow seasons is an effective strategy to promote resource utilization. The objective of this research was to compare the effects of planting different forage crops on the yields and nutritive compositions of forage and soil properties of winter fallow paddy in southern China. Five forage crops, including alfalfa (*Medicago sativa*, AF), common vetch (*Vicia sativa*, CV), milk vetch (*Astragalus sinicus*, MV), smooth vetch (*Vicia villosa*, SV) and Italian ryegrass (*Lolium multiflorum*, IR), were planted by monoculture on the winter fallow paddy in 2017-2018 (season 1) and 2018-2019 (season 2), respectively. The dry matter yield of IR was significantly higher than those of AF, CV, SV and MV (*P*<0.05). The crude protein yield of IR was significantly higher than those of AF, CV and MV (*P*<0.05). The neutral detergent fiber and acid detergent fiber contents of CV, SV and IR were significantly lower than those of AF and MV (*P*<0.05). Forage crops significantly affected the culturable microbial population of soils (*P*<0.05). The bacteria, actinomyces and fungi numbers on IR were the highest, while azotobacter number was the lowest. The catalase, acid-phosphatase and invertase activities of IR soil were the lowest. The numbers of bacteria, actinomyces and fungi of IR soil were the highest. IR and SV were the best crops to obtain forage and improve the soil. When producers pursue higher forage yield, we recommend planting Italian ryegrass. If the producers want to improve soil characteristics, smooth vetch is the most suitable plant. These results provide useful information to rice growers for cropping management when growing forage crops (based on the yield and nutritional value) or green manure (based on improving the soil fertility) as an alternative to late rice harvest.

## 1 Introduction

Grasses grown in rotations with corn (*Zea mays*) or rice (*Oryza sativa*) provide a range of help to improve soil fertility and crop yield in arid (Li et al., 2021) or humid regions (Xu et al., 2021). In general, legumes can obtain nitrogen from the atmosphere, reducing the input costs for agricultural production (mainly N fertilizer input) (Jensen et al., 2020). Legumes can improve water use efficiency and economic benefits in the areas where crop rotation systems are simple or unique (Peng et al., 2022). Meanwhile, crop rotations also showed positive effects on microorganisms of soils (Venter et al., 2016). The contribution of grass productivity on microbial community was higher than that of soil variables, and microbial community was more related to grass productivity (Wang et al., 2023). For example, phosphate-solubilizing bacteria play a crucial role in the circulation of phosphorus and the transfer of phosphorus to plants, and in improving crop productivity (Estrada-Bonilla et al., 2021). Some research results show that planting grasses not only improves soil physical and chemical properties, but also increases the number of soil bacteria and fungi (Chen et al., 2012; Ma et al., 2023). However, in multiple crop rotation systems, producers need to consider more factors, such as the direct economic benefits of yield and nutritional value of forage (Xu et al., 2021).

The population accounts is 823 million in southern China, and the demand for animal products is large, while the availability of feedstocks usually restricts the development of livestock production systems. In recent seasons, to provide more feedstocks, various forage crops, e.g., Italian ryegrass (*Lolium multiflorum*, IR), barley (*Hordeum vulgare*), wheat (*Triticum aestivum*), oat (*Avena sativa*), alfalfa (*Medicago sativa*, AF), common vetch (*Vicia sativa*, VS), milk vetch (*Astragalus sinicus*, MV) and smooth vetch (*Vicia villosa*, SV), have been introduced into the traditional early rice-late rice-winter fallow system, and the triple cropping system has been widely adopted and recommended. Similar rotation systems have also been recommended in Japan based on their proven advantages in economic return (Li et al., 2019). On the other hand, farmers also improve soil fertility by growing these crops. However, in addition to considering the costs of seeds, labors and plowing, the impact of planting forage crops during the fallow season on soil nutrients was an important aspect that producers should consider. There was currently very limited information on the soil impact of early rice-late rice forage crops rotation systems. Against this complex background, producers have been considering which forage crops will achieve both economic and ecological benefits.

At present, few researchers have focused on the forage yield and nutritive value in cropping systems. Despite these findings, uncertainty remains regarding how changes in forage yield and soil properties respond to intensifying double-rice cropping systems with different winter forage crops. In southern China, the advantages of different crop rotation systems (cereal crops in the summer and cash crops in the winter) have been revealed (Xian et al., 2023), but studies integrating forage into farming systems and scientifically evaluating the economic and ecological potential were still lacking. This study offers more options for overlooked feed producers, including: 1) selecting high-yield and high-quality forage crops that grow in the winter fallow fields; 2) choosing green manure crops that improve soil fertility. We hypothesize that 1) Different forage crops have different effects on soil characteristics and yields in the winter fallow fields; 2) there were positive correlations between soil microbial population and forage yield or nutritive value.

## 2 Materials and methods

### 2.1 Field experiment

The field experiment was conducted from Nov. 2017 to Mar. 2019 (after the late rice was harvested and before the early rice was planted) at the Experimental Field of South China Agricultural University, which is located in Ningxi (22°23′95″N, 113°63′27″E), Zengcheng district, Guangdong province, China. In the last 20 seasons, the monthly mean temperature from Jan. to Dec. was 22.4°C, and the monthly mean precipitation was 150 mm. The main soil characteristics of the field before the experiment were as follows: pH 5.65, organic matter 22.0 g kg^-1^, total nitrogen 1.01 g kg^-1^, available nitrogen 126 mg kg^-1^ and available phosphorus 13.2 mg kg^-1^. The total rainfall during the growing periods of forage crops in 2017-2018 (season 1) and 2018-2019 (season 2) were 388 mm and 286 mm (total rainfall in five months), respectively (**Table 1**). The annual average air temperature in season 1 and season 2 were 16.9°C and 17.0°C (mean of the five months), respectively. Paddy fields after harvesting late rice (Jul. planting, Nov. harvest) were used to grow five forage crops (to be harvested in spring next season). Subsequently, the early rice was planted (Apr. planting, Jul. harvest) (**Table 2**).

**Table 1:** Meteorological data during growing period of forage crops at the experimental fields.

Month	20-season average		Season 2		Season 1	
	Mean rainfall (mm)	Mean temperature (°C)	Mean rainfall (mm)	Mean temperature (°C)	Mean rainfall (mm)	Mean temperature (°C)
November	46	20.4	34.4	20.6	59	19.9
December	50	15.6	19.5	16.1	10	16.6
January	56	14.4	3.80	15.4	136	14.1
February	22	15.2	92.5	18.5	12	14.5
March	116	18.7	238	19.7	69	19.9

**Table 2:** Agricultural operations dates of rice.

Seasons	Crop types	Transplanting density of rice (10^4^/ha)	Sowing date	Base fertilizer date	Jointing fertilizer date	Harvesting date	Crop growth time (d)
Season 1	Early rice	14.4	1. Apr. 2017	10. Apr. 2017	22. May. 2017	26. Jul. 2017	117
	Late rice	14.4	30. Jul. 2017	8. Aug. 2017	20. Sep. 2017	15. Nov. 2017	109
Season 2	Early rice	14.4	4. Apr. 2018	15. Apr. 2018	24. May. 2016	25. Jul. 2018	113
	Late rice	14.4	28. Jul. 2018	5. Aug. 2018	25. Sep. 2016	10. Nov. 2018	106

### 2.2 Experimental treatments

Five forage crops, IR, AF, CV, milk vetch MV and SV, were arranged in a randomized block design with three replicates. Each plot was 3.0 by 4.0 m in size and was separated by 0.4 m walkways. The fields were planted with early rice from Apr. to Jul., and next planted by late rice from late Aug. to early Nov. The land after late rice harvest was rotary-plowed (JLGJ4.5, Zhejiang Taizhou Food Instrument Factory, Taizhou, China). The compound fertilizer (mixture fertilizers, N: P_2_O_5_: K_2_O = 15: 6: 8) was split-applied at the rate of 150 kg ha^-1^. Forty percent was applied as a basal fertilizer before sowing, and the remaining was applied for 52 to 54 days after seedling emergence. AF, CV, MV, SV and IR were sown at seeding rates of 22.5, 60.0, 75.0, 75.0 and 22.0 kg ha^-1^, respectively. All forage crops were broadcast. In this study, no insecticides and fungicides were used during the forage crops growing periods, and the experiments were conducted for two seasons (season 1 and season 2).

### 2.3 Forage crops and soil sampling

Forage crop and soil samples from the each plot were taken on 126 d (season 1) and 132 d (season 2) after sowing (**Table 3**). Five soil cores from 0-20 cm soil layer (diameter: 5 cm) that were distributed evenly across each plot were collected. The five soil cores were composited together into a single sample. Fresh soil samples were used to determine amounts of microorganisms. The remaining soil samples were air-dried in the laboratory, and soils were then sieved using a 2 mm mesh and analyzed for chemical components and enzymatic activities.

**Table 3:** Agricultural operations dates of forage crops.

Seasons	Crop types	Sowing rate (kg/ha)	Sowing date	First fertilization date	Second fertilization date	Growth stage	Harvesting date	Growth period (d)
Season 1	AF	22.5	22-Nov	2-Dec	26-Jan	Early blooming	27-Mar	126
	CV	60.0	22-Nov	2-Dec	26-Jan	Full blooming	27-Mar	126
	MV	75.0	22-Nov	2-Dec	26-Jan	Early blooming	27-Mar	126
	SV	75.0	22-Nov	2-Dec	26-Jan	Full blooming	27-Mar	126
	IR	22.0	22-Nov	2-Dec	26-Jan	Emergence of inflorescence completed	27-Mar	126
Season 2	AF	22.5	17-Nov	4-Dec	28-Jan	Early blooming	29-Mar	132
	CV	60.0	17-Nov	4-Dec	28-Jan	Full blooming	29-Mar	132
	MV	75.0	17-Nov	4-Dec	28-Jan	Early blooming	29-Mar	132
	SV	75.0	17-Nov	4-Dec	28-Jan	Full blooming	29-Mar	132
	IR	22.0	17-Nov	4-Dec	28-Jan	Emergence of inflorescence completed	29-Mar	132

AF, alfalfa; CV, common vetch; MV, milk vetch; SV, smooth vetch; IR, Italian ryegrass.

In each plot, 30 plants were randomly selected to measure plant height, an area of 1 m × 1 m was randomly selected to harvest crops, and the yield of fresh crops was determined. Then, 2 kg of fresh crop from each plot was taken back to the laboratory and dried at 70°C for 48 h to determine the dry matter (DM) content. The DM yield was calculated from dry matter content and fresh yield.

### 2.4 Microbial and chemical analyses

The dried crop samples were crushed and passed through a 1.0 mm sieve to determine the chemical compositions. Crude protein (CP, obtained by multiplying total nitrogen with a factor of 6.25) or total nitrogen, ether extract (AOAC, 1990) and water-soluble carbohydrates (WSC) (Murphy, 1958) contents were determined by Kjeldahl (Nitrogen analyzer KN680, Shandong Jinan Alva Instrument, Jinan, China), anhydrous ether and sulfuric acid-anthrone methods, respectively. The crude ash content was determined by burning in a muffle furnace at 550°C for 5 hours. Crude fiber, neutral detergent fiber (NDF), acid detergent fiber (ADF), and acid detergent lignin (ADL) concentrations were determined by a filter bag method (Ankom Technology, Macedon, NY), with heat-stable amylase and sodium sulfite used for NDF analysis (Van Soest et al., 1991). Hemicellulose content was calculated from the difference between NDF and ADF contents.

The DM partial productivity (DMPP), CP partial productivity (CPPP) and nitrogen use efficiency (NUE) were calculated according to the ratio of dry matter yield and nitrogen content to nitrogen fertilizer input (Yang et al., 2018). Relative feeding value (RFV) was calculated based on the ADF and NDF contents of crops (Rohweder et al., 1978).

Soils and distilled water were mixed at a ratio of 5 g: 25 mL and left to stand for 30 min to determine pH of soils (LE438 pH meter, Mettler Toldeo, Shanghai, China). Organic matter (Sims and Haby, 1971) and available phosphorus (Tran et al., 1988) contents of soils were determined by oxidative titration and isotope dilution methods, respectively. We determined soil enzymatic activity using the methods described by Guan (1986). Urease activity was determined with pH 6.7 citrate acid buffer solution at 37°C for 24 hours. Catalase activity was determined with the potassium permanganate titration after shaking soil sample at 25°C for 20 min. Acid-phosphatase activity was determined with P-nitrophenyl phosphate disodium at 37°C for 24 hours. Invertase activity was determined with 3, 5-dinitro-salicylic acid colorimetry at 37°C for 24 hours. In this study, 10 g fresh material (sterilized saline solution 90 mL, 8.5 g L^-1^ NaCl) were used to determined the culturable microbial population (Chen et al., 2021). The numbers of bacteria, actinomyces, fungi and azotobacter were counted by using nutrient agar, starch nitrate medium, potato dextrose agar, and nitrogen-fixing rhizobia agar, respectively. Aerobic bacteria were cultured at 37°C for 2 days under aerobic conditions. Actinomyces, fungi and azotobacter were cultured at 37°C for 3 days under aerobic conditions.

### 2.5 Data analyses

To test the effects of seasons, crops and their interactions on the yield and nutritive value of crop and soil chemical properties, repeated measures ANOVA (Duncan’s multiple range test) was used. The normality and homogeneity of variance were checked using statistical tests. If the p-value associated with these tests was less than a pre-determined significance level (0.05), then we rejected the null hypothesis, indicating that the variances was not equal across groups. Conversely, if the P-value was greater than the significance level, we failed to reject the null hypothesis, suggesting that the variances was homogeneous. Pearson correlation (normality and homogeneity of Pearson residuals were checked firstly) was used to determine the relationships among yields, plant heights and nutritional compositions of crops or chemical properties, enzyme activities and microorganisms of soils (SPSS 25.0 for Windows; SPSS, Chicago, IL). The model used was:


$$Y=\mu +T_{i}+\varepsilon_{ij}$$

where Y was the response variable, μ was the overall mean, T_i_ was the effect of treatment, and ε_ij_ was the residual error.

To further investigate the pattern of relationships among the indicators (all measured variable), principal component analysis (PCA) was performed on two data sets of (1) forage yield and nutritional compositions and (2) soil chemical properties and microbial population (OriginPro®2022b software, OriginLab Corp., W A, USA). Based on the information of grass yield and nutrient composition and soil characteristics, the fuzzy membership function analysis method was used to sort the five forage crops.

## 3 Results

### 3.1 Yield, plant height and partial productivity of crops

Forage crop species had significant effects on the yield, plant height, DMPP, CPPP and NUE (*P*<0.001). seasons had significant effects on the CP yield, plant height, RFV and CPPP of crops (*P*<0.05). The interaction of seasons and crop species on the plant height was significant (*P*<0.001) (**Table 4**). Compared to season 1, the DM and CP yields in season 2 decreased from 3.74 t ha^-1^ to 2.99 t ha^-1^ (25.1%) and 0.59 t ha^-1^ to 0.42 t ha^-1^ (40.5%), respectively. The plant height and RFV in season 2 were significantly higher than those in season 1. IR had the highest DM yield (7.33 t/ha), CP yield (0.70 t/ha), DM partial productivity (81.5 kg/kg), CP partial productivity (7.73 kg/kg), and NUE (67.1 kg/kg) among the forage crops species. The yields and partial productivity of AF and MV were significantly lower than those SV and IR (*P*<0.05), while their RFV was higher (*P*<0.05). The DM yield and DMPP of IR were significantly higher than those of other four forage crops (*P*<0.05), while the CP yield and CPPP of SV was not different from IR.

**Table 4:** Plant height, yield and relative feed value for different seasons and forage crops.

Seasons and crop types		Dry matter yield (t/ha)	Crude protein yield (t/ha)	Plant height (cm)	Relative feed value	Dry matter partial productivity (kg/kg)	Crude protein partial productivity (kg/kg)	Nitrogen use efficiency (kg/kg)
Seasons (Y)	Season 1	3.74 ± 0.65	0.59 ± 0.06a	43.3 ± 3.76a	124 ± 4.18a	41.6 ± 7.22	6.55 ± 0.61a	37.3 ± 4.25
	Season 2	2.99 ± 0.53	0.42 ± 0.04b	29.4 ± 4.26b	108 ± 2.96b	33.2 ± 5.85	4.64 ± 0.46b	41.9 ± 3.91
	Average value	3.36	0.50	36.4	116	37.4	5.60	39.6
	Quadratic sum	4.26	0.22	1468	1954	525	27.6	162
	*F*	0.81	6.22	6.06	9.91	0.81	6.24	0.65
Crops (C)	AF	1.30 ± 0.15c	0.27 ± 0.04d	30.7 ± 5.50b	130 ± 5.86a	14.5 ± 1.63c	3.01 ± 0.50d	31.4 ± 1.95b
	CV	2.78 ± 0.32b	0.52 ± 0.05bc	30.9 ± 2.84b	105 ± 2.78b	30.9 ± 3.52b	5.75 ± 0.53bc	34.5 ± 4.82b
	MV	1.76 ± 0.10c	0.38 ± 0.04cd	14.9 ± 2.90c	132 ± 4.69a	19.6 ± 1.09c	4.19 ± 0.39cd	29.9 ± 1.74b
	SV	3.64 ± 0.31b	0.66 ± 0.07ab	54.1 ± 4.02a	103 ± 3.00b	40.4 ± 3.48b	7.30 ± 0.80ab	35.2 ± 1.33b
	IR	7.33 ± 0.46a	0.70 ± 0.06a	51.1 ± 1.22a	109 ± 4.21b	81.5 ± 5.10a	7.73 ± 0.69a	67.1 ± 3.79a
	Average value	3.36	0.50	36.4	116	37.4	5.60	39.6
	Quadratic sum	138	0.78	6321	4751	17024	97.0	5774
	*F*	65.1	11.1	20.5	10.9	65.2	11.2	26.1
*P* value	Y	0.375	0.019	0.020	0.004	0.376	0.019	0.427
	C	0.000	0.000	0.000	0.000	0.000	0.000	0.000
	Y × C	0.330	0.124	0.000	0.072	0.327	0.130	0.541
	Quadratic sum (Y × C)	1.77	0.06	350	259	219	7.68	168
	*F* (Y × C)	1.23	2.06	15.5	2.54	1.24	2.02	0.80

AF, alfalfa; CV, common vetch; MV, milk vetch; SV, smooth vetch; IR, Italian ryegrass. Different lowercase letters in the same column represent significant differences between experiment seasons or crops (*P*< 0.05). Quadratic sum means the sum of squares among groups.

### 3.2 Nutritional composition of crops

Crop species had no effects on crude fiber and crude ash contents, but significantly affected other nutritional indicators (*P*<0.05 or 0.01) (**Table 5**). The ether extract, crude fiber, crude ash, NDF, ADF and WSC contents of crops were different between the seasons (*P*<0.05). The DM and CP contents of crops varied from 161 to 173 g kg^-1^ and 162 to 191 g k^-1^g in two seasons, respectively (**Table 6**). Compared to season 1, an obvious decrease in ether extract and WSC contents in season 2 were detected (*P*<0.05). The ADF and NDF contents of CV, SV and IR were significantly higher than those of AF and MV (*P*<0.05). The ADL content of IR was lower than other four crops, whereas the content of WSC was higher than other four crops (*P*<0.05).

**Table 5:** Nutritional composition of forage crops in different seasons.

Seasons and crop types		Dry matter (g/kg, FM)	Nutritional composition (g/kg DM)								
			Crude protein	Ether extract	Crude fiber	Crude ash	NDF	ADF	Hemicellulose	WSC	ADL
Seasons (Y)	Season 1	173 ± 5.80	191 ± 15.4	49.0 ± 4.21a	200 ± 7.78b	87.5 ± 4.18b	511 ± 10.6b	276 ± 12.3b	235 ± 7.37	81.1 ± 9.07a	174 ± 13.2
	Season 2	161 ± 3.76	162 ± 10.3	38.5 ± 2.58b	242 ± 6.72a	119 ± 3.64a	561 ± 7.96a	311 ± 11.5a	250 ± 6.44	51.6 ± 4.84b	207 ± 11.2
	Average value	167	177	43.7	221	103	536	293	242	66.5	190
	Quadratic sum	1040	6327	823	13078	7588	19031	9155	1787	6671	8168
	*F*	2.90	2.46	4.51	16.5	32.9	14.5	4.31	2.49	8.42	3.65
Crops (C)	AF	183 ± 7.55a	203 ± 12.8a	53.7 ± 4.24ab	250 ± 14.2	101 ± 8.47	499 ± 10.2b	250 ± 8.89b	249 ± 11.3ab	57.9 ± 6.32bc	190 ± 9.47b
	CV	173 ± 2.73ab	195 ± 20.3a	36.9 ± 1.96c	223 ± 14.4	117 ± 9.76	570 ± 8.92a	315 ± 8.95a	256 ± 4.27ab	49.6 ± 5.51bc	222 ± 14.9a
	MV	140 ± 2.47c	212 ± 12.6a	56.1 ± 6.54a	214 ± 11.6	112 ± 6.45	499 ± 13.3b	237 ± 10.9b	262 ± 11.8a	72.0 ± 5.23b	210 ± 11.0a
	SV	162 ± 4.24b	179 ± 6.74a	29.7 ± 3.91c	217 ± 14.7	96.4 ± 7.05	561 ± 10.3a	343 ± 10.9a	217 ± 8.92c	41.2 ± 4.99c	218 ± 9.99a
	IR	175 ± 7.25ab	94.7 ± 5.55b	42.2 ± 4.30bc	199 ± 11.4	90.7 ± 10.7	550 ± 14.9a	321 ± 11.3a	229 ± 8.81bc	112 ± 14.9a	109 ± 9.07c
	Average value	167	177	43.7	221	103	536	293	242	66.50	190
	Quadratic sum	6844	54140	2990	8540	2837	28261	52895	8693	18517	52459
	*F*	10.1	13.9	6.34	2.00	1.58	6.43	21.0	4.11	11.2	17.8
*P* value	Y	0.100	0.128	0.043	0.000	0.000	0.001	0.047	0.126	0.007	0.066
	C	0.000	0.000	0.000	0.126	0.210	0.001	0.000	0.011	0.000	0.000
	Y × C	0.030	0.362	0.062	0.559	0.157	0.184	0.649	0.512	0.000	0.980
	Quadratic sum (Y × C)	1276	3362	740	1816	979	2164	731	1655	2361	210
	*F* (Y × C)	3.33	1.15	2.67	0.77	1.86	1.72	0.63	0.85	8.98	0.11

AF, alfalfa; CV, common vetch; MV, milk vetch; SV, smooth vetch; IR, Italian ryegrass. Different lowercase letters in the same column represent significant differences between experiment seasons or crops (*P*< 0.05). DM, dry matter; FM, fresh matter. Quadratic sum means the sum of squares among groups.

**Table 6:** Soil chemical properties grown with various forage crops in different seasons.

Seasons and crop types		pH	Available phosphorus (mg/kg)	Organic matter (mg/kg)	Total nitrogen (mg/kg)
Seasons (Y)	Season 1	5.06 ± 0.03b	23.1 ± 0.44a	20.5 ± 0.32b	1.50 ± 0.07a
	Season 2	5.56 ± 0.05a	16.9 ± 0.49b	22.3 ± 0.27a	1.27 ± 0.03b
	Average value	5.31	20.0	21.4	1.38
	Quadratic sum	1.92	283	22.5	0.38
	*F*	65.6	86.0	17.4	9.53
Crops (C)	AF	5.32 ± 0.18	19.7 ± 1.85	20.1 ± 0.68b	1.30 ± 0.08
	CV	5.30 ± 0.09	19.5 ± 1.40	22.0 ± 0.18a	1.45 ± 0.13
	MV	5.24 ± 0.16	20.7 ± 0.70	21.4 ± 0.52ab	1.35 ± 0.05
	SV	5.41 ± 0.13	21.9 ± 1.77	21.0 ± 0.43ab	1.56 ± 0.10
	IR	5.20 ± 0.05	18.3 ± 1.45	22.5 ± 0.57a	1.28 ± 0.06
	Average value	5.31	20.0	21.4	1.38
	Quadratic sum	0.15	42.0	20.3	0.32
	*F*	0.35	0.79	3.31	1.68
*P* value	Y	0.000	0.000	0.000	0.005
	C	0.840	0.544	0.026	0.187
	Y × C	0.000	0.000	0.019	0.000
	Quadratic sum (Y × C)	0.45	32.0	6.86	0.52
	*F* (Y × C)	9.94	8.87	3.80	9.00

AF, alfalfa; CV, common vetch; MV, milk vetch; SV, smooth vetch; IR, Italian ryegrass. Different lowercase letters in the same column represent significant difference between experiment seasons or crops (*P*< 0.05). Quadratic sum means the sum of squares among groups.

### 3.3 Chemical properties, enzyme activities and microorganisms of soils

The interaction of seasons and crop species affected pH value, available phosphorus, organic matter and total nitrogen contents of soils (*P*<0.05 or *P*<0.001). The pH value and organic matter content of soils in season 1 were significantly lower than those in season 2 (*P*<0.05), while the available phosphorus and total nitrogen contents were higher in season 1. The organic matter content of AF soil was lower than that of CV and IR soils (*P*<0.05).

The urease, catalase, and invertase activities in season 2 were lower than those in season 1 (*P*<0.001, **Supplementary Table S1**). Crop species had slight effects on enzyme activity of soils, and had significant effects on microbial numbers of soils (*P*<0.05). The acid-phosphatase activity of IR (67.7 mg/g/24h) soil was lower than that of AF (335 mg/g/24h), CV (332 mg/g/24h), MV (332 mg/g/24h) and SV (358 mg/g/24h) soils (*P*<0.05). Crop species had significant effects on microbial numbers of soils (*P*<0.05). The numbers of soil microorganisms (except for bacteria) in season 1 were more than in season 2. Compared to other four crops, the bacteria (6.81 lg cfu/g FM), actinomyces (5.77 lg cfu/g FM) and fungi (5.28 lg cfu/g FM) numbers of IR soils were the highest, while azotobacter (3.70 lg cfu/g FM) numbers was the lowest.

### 3.4 Interrelation pattern among crops or soil quality indicators

**Figure 1:**
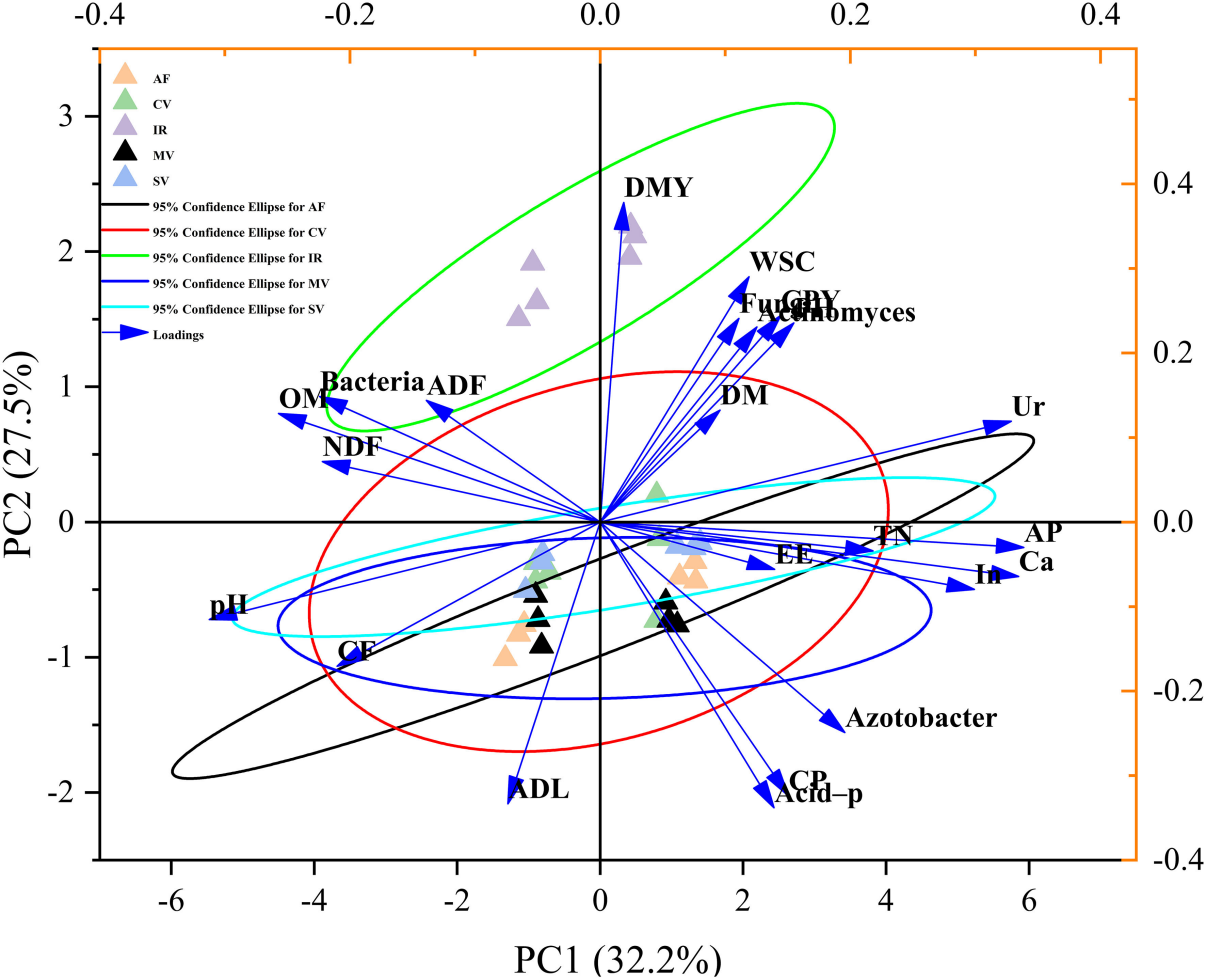
Projection on the first two principal components on the basis of crops and soils. The first axis accounts for 32.2% of the total variance and the second for 27.5%. Also inserted are the original attributes, with their vectors intersecting at (0, 0). The length of each attribute vector is proportional to its contribution to the principal component axis. The ellipse indicates 95% confidence. DMY, dry matter yield; CPY, crude protein yield; DM, dry matter; CP, crude protein; EE, ether extract; CF, crude fiber; NDF, neutral detergent fiber; ADF, acid detergent fiber; WSC, water-soluble carbohydrates; ADL, acid detergent lignin; AP, available phosphorus; OM, organic matter; TN, total nitrogen; Ur, urease; Ca, catalase; Acid-p, acid-phosphatase; In, invertase. AF, alfalfa; CV, common vetch; MV, milk vetch; SV, smooth vetch; IR, Italian ryegrass.

In terms of correlation, there were negative correlations between CP and WSC contents, between ADF or NDF and ether extract contents, between WSC and crude fiber or ADL contents. There were positive correlations between organic matter content and bacterial numbers, between total nitrogen content and four enzymatic activities (**Supplementary Table S1**). In the PCA, the cumulative contribution rates of PC1 and PC2 was 59.5%. Specifically, IR had the highest yield and WSC content (**Tables 4**, **5**), which was clearly separated from the other four crops along the second axis (**Figure 1**). On the other hand, IR had the most bacteria, actinomyces (**Table 7**), and fungi, which was clearly separated from the other four crops along the first axis (**Figure 1**). However, there were no differences in most nutrients of four legume forage crops and their effects on most soil nutrients and enzyme activity. This was also the reason that the 95% confidence intervals overlap. According to the results of the membership function analysis, SV scored the highest, while AF scored the lowest, and was not affected by the season (**Table 8**).

**Table 7:** Enzyme activities and microorganisms of soils grown with various forage crops in different seasons.

Seasons and crop types		Enzymatic activities				Microorganisms (lg cfu/g FM)			
		Urease (mg/g/24 h)	Catalase (mg/g/20 min)	Acid-phosphatase (mg/g/24 h)	Invertase (mg/g/24 h)	Bacteria	Actinomyces	Fungi	Azotobacter
Seasons (Y)	Season 1	0.50 ± 0.01a	5.01 ± 0.39a	314 ± 34.1	21.3 ± 1.79a	6.14 ± 0.09b	5.52 ± 0.10a	4.92 ± 0.06	5.54 ± 1.92a
	Season 2	0.21 ± 0.01b	1.38 ± 0.11b	256 ± 27.1	10.1 ± 0.50b	6.65 ± 0.10a	5.20 ± 0.11b	4.59 ± 0.16	4.69 ± 0.23b
	Average value	0.35	3.20	285	15.7	6.39	5.36	4.75	5.11
	Quadratic sum	0.64	98.5	25008	935	1.98	0.77	0.79	5.39
	*F*	225	79.0	1.75	36.2	14.7	4.91	3.66	6.81
Crops (C)	AF	0.33 ± 0.09	3.15 ± 0.83	335 ± 17.8a	12.2 ± 1.92	6.24 ± 0.08b	5.33 ± 0.23bc	4.58 ± 0.23b	5.07 ± 0.33b
	CV	0.35 ± 0.07	3.36 ± 1.02	332 ± 25.3a	15.8 ± 2.35	6.13 ± 0.13b	5.61 ± 0.10ab	4.70 ± 0.23b	5.93 ± 0.26a
	MV	0.36 ± 0.07	3.23 ± 0.84	332 ± 32.2a	17.9 ± 3.34	6.60 ± 0.24ab	5.09 ± 0.06c	4.66 ± 0.19b	5.34 ± 0.11ab
	SV	0.32 ± 0.05	4.37 ± 1.11	358 ± 15.3a	21.8 ± 4.24	6.19 ± 0.14b	5.00 ± 0.13c	4.55 ± 0.10b	5.54 ± 0.32ab
	IR	0.42 ± 0.06	1.88 ± 0.38	67.7 ± 3.79b	10.9 ± 1.23	6.81 ± 0.14a	5.77 ± 0.04a	5.28 ± 0.09a	3.70 ± 0.22c
	Average value	0.36	3.20	285	15.7	6.40	5.36	4.75	5.11
	Quadratic sum	0.03	18.8	356897	463	2.08	2.65	2.12	17.4
	*F*	0.31	1.03	33.1	2.42	3.55	6.63	2.83	10.7
*P* value	Y	0.000	0.000	0.196	0.000	0.001	0.035	0.066	0.014
	C	0.868	0.413	0.000	0.075	0.020	0.001	0.046	0.000
	Y × C	0.022	0.002	0.107	0.000	0.114	0.000	0.298	0.297
	Quadratic sum (Y × C)	0.02	8.94	12919	167	0.50	1.14	0.81	1.00
	*F* (Y × C)	3.63	6.25	2.19	8.96	2.14	9.74	1.32	1.32

AF, alfalfa; CV, common vetch; MV, milk vetch; SV, smooth vetch; IR, Italian ryegrass. Different lowercase letters in the same column represent significant difference between experiment seasons or crops (*P* < 0.05). Quadratic sum means the sum of squares among groups.

**Table 8:** Scores and rankings of forage crops in different seasons.

Seasons	Crop type	Composite scores	Ranking in composite scores
Season 1	Alfalfa	0.547	5
	Common vetch	0.617	2
	Milk vetch	0.548	4
	Smooth vetch	0.768	1
	Italian ryegrass	0.560	3
Season 2	Alfalfa	0.182	10
	Common vetch	0.318	8
	Milk vetch	0.269	9
	Smooth vetch	0.404	6
	Italian ryegrass	0.355	7

## 4 Discussion

In this study, as the five crops were commonly used as green manure or forage crops of winter fallow paddy in southern China, the research had certain scientific and practical value. The yield and plant height were greater in season 1 than in season 2. This was because the rainfall was more abundant and more intensive before the crops were harvested in season 2 (Mar.), and significantly affected the growth of crops. When the moisture content of soils was high, the rapid depletion of oxygen and slow diffusion in the soil would lead to hypoxia in plant roots. Moreover, the aerenchyma of roots also changed accordingly and determines the movement of oxygen in the root system by adjusting the porosity when soil moisture content was high (Colmer, 2003). However, the eventual result is a decrease in the yield of grass.

In general, plants attempt to cope with hypoxic conditions by regulating metabolic activities, such as ethylene synthesis, carbohydrate breakdown, glycolysis and ethanol fermentation (Perata, 2020). Plants could also generate energy by fermenting WSC to withstand waterlogging stress (Moura et al., 2010). However, the WSC content of crops was very limited in this study (**Table 5**). Due to excessive rainfall in season 2, the WSC content was lower than in season 1 (waterlogging stress). AF had the lowest DM yield, since its roots had lower porosity and the arrangement of cells in the cortex is hexagonal, so they were most sensitive to waterlogging (Zook et al., 1986). On the other hand, oxygen deficiency inhibits the uptake of nutrients by roots of crops, which inevitably leads to nutrient deficiencies in the shoots. As observed in this study, the CP content and some other soluble components accumulated and caused the cell wall to be diluted in season 1 (cellular contents were diluted), thus the crude fiber, ADF and NDF contents were lower than those in season 2. It was well-known that AF was more sensitive to the pH of soil compared to the other four crops (Blazinkov et al., 2008). The soils showed obviously weak acidity in this study, which was another reason for the lowest DM and CP yields of AF. In this study, all the crops were harvested before planting the early rice. It was worth noting that AF and MV were important plants because of their perennial and biennial characteristics, especially alfalfa, but it might a low yield in the first season and reaches its highest yield after the second season. It was not logical to compare the yields of annuals and perennials, and the other indicators take into account the results of only two seasons. This was one of the shortcomings of this study.

Generally, due to genetic and morphological limitations of harvest (crop performance), there are differences in yield and quality indicators of crops (e.g. leaf morphological structures, leaf senescence and sink control), some nutritional indicators of different crops were different (Zhang et al., 2018). During plant growth, photosynthetic products temporarily converge mainly in the stem before flowering, followed by leaves (low ratio of leaves to whole plant). As a result MV had the lowest plant height in this study, and the lowest DM content. On the other hand, the increase in photosynthetic products of cell wall also increased the DM content. Through a symbiotic relationship between plant and bacteria of soils, legume species could fix nitrogen from atmospheric (Plaza-Bonilla et al., 2015). This explains why the CP content of IR was the lowest in the present study. Some studies had observed that the fiber content of grasses was higher than legumes (Ergon et al., 2017). However, this phenomenon was not obvious in this study, which might be related to the stems and leaves ratio of crops. Unfortunately, morphological data (except for plant height) were not collected from the forage species at harvest in this study. The WSC content was different among four legumes in this study (*P*<0.05), and the WSC content of IR was higher than four legumes (*P*<0.05). The reason was that more WSC could be stored in the mesophyll of IR.

The ADF and NDF contents of AF and MV were significantly lower than those of CV and SV (*P*<0.05). This was closely related to the maturity of crops (**Table 3**). In general, increasing maturity increased NDF, ADF and ADL contents of herbage, and reduced CP content (**Tables 3**, **5**). The data on harvest stage differences among five forage crops also explain maturity differences (**Table 3**). This explains the low RFV of CV and SV in this study. The concentrations of all fibrous components (NDF, ADF, hemicellulose, cellulose and lignin) in grasses typically increase and digestibility of nutrients decreases with maturity (Rinne et al., 1997). Therefore, higher ADF and NDF content have a negative impact on milk production (Günal et al., 2019) and body weight (Sousa et al., 2017) in ruminant animals. As a result, AF and MV would improve milk production and body weight more than the other three crops in this study. However, producers should consider the nutrient supply of each forage for animals and the potential hazards of nitrate concentration in legume forage. The NDF content in legumes below 400 g/kg DM is considered good in USA, whereas NDF content up to about 500 g/kg DM in grasses is considered desirable. Forages with ADF over 350 g/kg DM are considered low quality. This indicates that the nutritional value of the forage in this study was relatively low. Compared with the traditional early rice-late rice-fallow system, the early rice-late rice-fallow forage crops rotation systems improves the productivity of the winter paddy field and increases the forage supply of the farm. Therefore, rice growers were willing to adopt the rice forage production systems. There are similar conclusions in Japan (Li et al., 2019). It was worth noting that a field experimental based data related to forage productivity, nutritent quality and the scale of rice cultivation related to animal feeding time information should be determined, which facilitates the maximization of economic benefits in early rice-late rice- forage crops rotation systems. Unfortunately, this study did not investigate the production performance of animals fed with five forage crops.

Usually, including forage crops within rotation system would significantly increase soil’s organic carbon content, and legumes have greater organic carbon of soils than grasses (Plaza-Bonilla et al., 2015; Sainju et al., 2015). However, this phenomenon was not detected in this study. The study field was mainly composed of fine soil for planting rice, with suitable moisture content and low pH, which was more suitable for the growth of the crops except AF. In particular, the roots (important source of soil organic matter) of IR have a high biomass mainly distributed in shallow soils, the highest soil organic matter content (Zhang and Zhou, 2019). The contribution of IR to soil organic matter was greater over a limited time. In this study, there was more rainfall during Feb. and Mar. in season 2 than in season 1 (**Table 1**), we speculate that the organic matter traps the exchangeable aluminum and increases the pH of soil (**Table 6**). As the pH value of soil increases, the content of soil organic matter increases, the cation exchange capacity increases, and the number of soil microorganisms increases, which was beneficial to the improvement of soil nutrient cycling capacity (Ye et al., 2023). Therefore, there was a significant positively correlated between bacteria number and organic matter content (*P*<0.001). This was consistent with the results reported by Ye et al. (2023). The content of available phosphorus was negatively correlated with the content of soil organic matter (*P*<0.01) (**Supplementary Table S1**). The reason was that some of available phosphorus were absorbed and utilized by microorganisms. All of these factors have the potential to be factors in improving the yield of the subsequent crops. This was because, a large number of studies have shown that increasing the crop replanting index was beneficial to the yield of the subsequent crop (Li et al., 2018; Xian et al., 2023).

It seems the interaction effect of seasons and crop species by crop species on soil properties due to confounding effect because the crops did not significantly affect the chemical properties of soils (except for organic matter content and acid-phosphatase activity). Previous studies had shown that soil enzyme activity was strongly influenced by crop species (Singh et al., 2021). However, crop species only affected acid-phosphatase activity in this study (*P*<0.05). Usually, the growth of leguminous crops stimulates the secretion of organic anions and acid phosphatase, which leaded to an increase in acid-phosphatase activity in the soil. This was consistent with the results reported by Sun et al. (2020). The effectiveness of potential soil nutrients (available N and P) has a high regulatory and controlling effect (positively correlated) on soil enzyme activity. In particular, AF had higher requirements for drainage of soils. As a result, the number of azotobacter in AF soils was lower than that in other three legumes soils (affected by rainfall) (**Table 7**). Winter fallow fields in this aera were not suitable for the growth of AF root systems and the multiplication of azotobacter on this basis. Although IR soils had the smallest number of azotobacter, it had the largest numbers of bacteria, actinomycetes and fungi. This might be related to the contribution of root metabolites to soil organic carbon. On the other hand, the differences in the composition and decomposition rate of plant roots also influence the numbers of microorganisms (De Graaff et al., 2010).

Some plants have a higher ability to acquire nutrients in soils rich in specific nutrients (mainly due to the extension of microorganisms on the roots). In general, lignin content changes in response to a plant’s soil microbial community (Bennett et al., 2015). Rhizobia and mycorrhizal fungi affect the transport of plant nutrients, while different proportions of plant nutrients were thought to influence the lignin synthesis pathway (Moura et al., 2010). Unfortunately, this study did not determine the effects of detailed functional microorganisms on lignin. In addition, microbially induced changes in the lignin synthesis pathway might also affect the nutritional value of crops (Moura et al., 2010). These theories can explain why the number of azotobacter was positively correlated with the total nitrogen content of soils or the CP content of crops in this study (*P*<0.05) (**Supplementary Table S1**). Unfortunately, because of the complex role of soil enzyme activity and microorganisms in nutrient cycling, researches on their effects on forage nutrients was limited in this study.

The results based on the analysis of the affiliation function showed that the seasons and crop types had a great impact on forage yield, nutrition and soil properties (**Table 8**). This was similar to the results of a number of studies (Weidhuner et al., 2022). It was clear that season 1 had a better impact on the agricultural system than that in season 2. Regardless of the season, SV had a better combined effect on improving forage yield and increasing soil fertility, with AF being the worst, compared to other three crops.

## 5 Conclusion

To reduce the fertilizer input in agricultural production, it is necessary to design an alternative nutrient management strategy. Cover crops can produce livestock forage and improve soil nutrient of winter fallow paddy in southern China, and forage had high feeding potential in early rice-late rice-forage crops rotation systems. When producers pursue higher forage yield, we recommend planting Italian ryegrass. If the producers want to improve soil characteristics, SV is the most suitable plant. These results provide useful information to rice growers for cropping management when growing forage crops (based on the yield and nutritional value) or green manure (based on improving the soil fertility) as an alternative to late rice harvest.

## Data availability statement

The raw data supporting the conclusions of this article will be made available by the authors, without undue reservation.

## Author contributions

LX: Data curation, Writing – original draft, Writing – review & editing. GT: Data curation, Investigation, Writing – review & editing. DW: Data curation, Investigation, Writing – review & editing. JZ: Conceptualization, Formal analysis, Funding acquisition, Methodology, Project administration, Resources, Writing – original draft, Writing – review & editing.

## References

[B1] AOAC (1990). Official methods of analysis. 15th ed (Arlington, VA: Association of Official Agricultural Chemists).

[B2] Bennett A. E. Grussu D. Kam J. Caul S. Halpin C. (2015). Plant lignin content altered by soil microbial community. New Phytol. 206, 166–174. doi: 10.1111/nph.13171 25389017

[B3] Blazinkov M. Sikora S. Macesic D. Uher D. Durakovic L. (2008). The effect of rhizobial inoculation and liming on alfalfa production in Croatia. Cereal Res. Commun. 36, 343–346.

[B4] Chen S. Zheng X. Wang D. Y. Chen L. P. Xu C. M. Zhang X. F. (2012). Effect of long-term paddy-upland seasonly rotations on rice (*Oryza sativa*) yield, soil properties, and bacteria community diversity. Sci. World J. 2012, 1–11. doi: 10.1100/2012/279641 PMC3417201 22919301

[B5] Chen X. Dong Z. Zhang J. (2021). Spraying sugars, growth temperatures and N application levels change epiphytic lactic acid bacteria composition on Italian ryegrass. Grassland Sci. 68, 145–154. doi: 10.1111/grs.12350

[B6] Colmer T. D. (2003). Long-distance transport of gases in plants: a perspective on internal aeration and radial oxygen loss from roots. Plant Cell Environ. 26, 17–36. doi: 10.1046/j.1365-3040.2003.00846.x

[B7] De Graaff M. A. Classen A. T. Castro H. F. SChadt C. W. (2010). Labile soil carbon inputs mediate the soil microbial community composition and plant residue decomposition rates. New Phytol. 188 (4), 1055–1064. doi: 10.1111/j.1469-8137.2010.03427.x 21058948

[B8] Ergon Å. Kirwan L. Fystro G. Bleken M. A. Collins R. P. Rognli O. A. (2017). Species interactions in a grassland mixture under low nitrogen fertilization and two cutting frequencies. II. Nutritional quality. Grass Forage Sci. 72, 333–342. doi: 10.1111/gfs.12257

[B9] Estrada-Bonilla G. A. Durrer A. Cardoso E. J. B. N. (2021). Use of compost and phosphate-solubilizing bacteria affect sugarcane mineral nutrition, phosphorus availability, and the soil bacterial community. Appl. Soil Ecol. 157, 103760. doi: 10.1016/j.apsoil.2020.103760

[B10] Guan S. Y. (1986). Soil enzymes and its methodology (Beijing: Agriculture Press).

[B11] Günal M. McCourt A. Zhao Y. Yan Z. G. Yan T. (2019). The effect of silage type on animal performance, energy utilisation and enteric methane emission in lactating dairy cows. Anim. Production Sci. 59 (3), 499–505. doi: 10.1071/AN16435

[B12] Jensen E. S. Carlsson G. Hauggaard-Nielsen H. (2020). Intercropping of grain legumes and cereals improves the use of soil N resources and reduces the requirement for synthetic fertilizer N: A global-scale analysis. Agron. Sustain. Dev. 40, 1–9. doi: 10.1007/s13593-020-0607-x

[B13] Li B. Ishii Y. Idota S. Tobisa M. Niimi M. Yang Y.. (2019). Yield and quality of forages in a triple cropping system in Southern Kyushu, Japan. Agronomy 9 (6), 277. doi: 10.3390/agronomy9060277

[B14] Li G. D. Schwenke G. D. Hayes R. Lowrie A. Lowrie R. Poile G.. (2021). Can legume species, crop residue management or no-till mitigate nitrous oxide emissions from a legume-wheat crop rotation in a semi-arid environment? Soil Tillage Res. 209, 104910. doi: 10.1016/j.still.2020.104910

[B15] Li X. B. Suo H. C. An K. Fang Z. W. Wang L. Zhang X. L.. (2018). The effect of mulching on soil temperature, winter potato (*Solanum Tuberosum* L.) growth and yield in field experiment, south China. Appl. Ecol. Environ. Res. 16, 913–929. doi: 10.15666/aeer/1602_913929

[B16] Ma X. T. Liao J. A. Zhao J. F. A. (2023). Meta-analysis of the effects on soil quality in Xinjiang (China) orchards after grass cultivation. Appl. Ecol. Environ. Res. 21 (3), 1891–1902. doi: 10.15666/aeer/2103_18911902

[B17] Moura J. C. Bonine C. A. de Oliveira Fernandes Viana J. Dornelas M. C. Mazzafera P. (2010). Abiotic and biotic stresses and changes in the lignin content and composition in plants. J. Integr. Plant Biol. 52, 360–376. doi: 10.1111/j.1744-7909.2010.00892.x 20377698

[B18] Murphy R. (1958). A method for the extraction of plant samples and the determination of total soluble carbohydrates. J. Sci. Food Agric. 9, 714–717. doi: 10.1002/jsfa.2740091104

[B19] Peng Y. Li Z. Sun T. Zhang F. Wu Q. Du M.. (2022). Modeling long-term water use and economic returns to optimize alfalfa-corn rotation in the corn belt of northeast China. Field Crops Res. 276, 1–14. doi: 10.1016/j.fcr.2021.108379

[B20] Perata P. (2020). Ethylene signaling controls fast oxygen sensing in plants. Trends Plant Sci. 25 (1), 3–6. doi: 10.1016/j.tplants.2019.10.010 31734094

[B21] Plaza-Bonilla D. Nolot J.-M. Raffaillac D. Justes E. (2015). Cover crops mitigate nitrate leaching in cropping systems including grain legumes: Field evidence and model simulations. Agriculture Ecosyst. Environ. 212, 1–12. doi: 10.1016/j.agee.2015.06.014

[B22] Rinne M. Huhtanen P. Jaakkola S. (1997). Grass maturity effects on cattle fed silage-based diets. 2. Cell wall digestibility, digestion and passage kinetics. Anim. Feed Sci. Technol. 67 (1), 19–35. doi: 10.1016/S0377-8401(96)01142-X

[B23] Rohweder D. A. Barnes R. F. Jorgensen N. (1978). Proposed hay grading standards based on laboratory analyses for evaluating quality. J. Anim. Sci. 47, 747–759. doi: 10.2527/jas1978.473747x

[B24] Sainju U. M. Singh H. P. Singh B. P. (2015). Cover crop effects on soil carbon and nitrogen under bioenergy sorghum crops. J. Soil Water Conserv. 70 (6), 410–417. doi: 10.2489/jswc.70.6.410

[B25] Sims J. R. Haby V. A. (1971). Simplified colorimetric determination of soil organic matter. Soil Sci. 112, 137–141. doi: 10.17221/185/2010-PSE

[B26] Singh S. R. Yadav P. Singh D. Bahadur L. Singh S. P. Yadav A. S.. (2021). Impact of different cropping systems on the land nutrient index, microbial diversity, and soil quality. Land Degradation Dev. 32, 3973–3991. doi: 10.1002/ldr.3863

[B27] Sousa D. O. Mesquita B. S. Pires A. V. Santana M. H. D. Silva L. F. P. (2017). Effects of fibre digestibility and level of roughage on performance and rumen fermentation of finishing beef cattle. Tropical. Anim. Health And Production 49 (7), 1503–1510. doi: 10.1007/s11250-017-1353-1 28712043

[B28] Sun B. Gao Y. Wu X. Ma H. Zhang C. Wang X.. (2020). The relative contributions of pH, organic anions, and phosphatase to rhizosphere soil phosphorus mobilization and crop phosphorus uptake in maize/alfalfa polyculture. Plant Soil 447, 117–133. doi: 10.1007/s11104-019-04110-0

[B29] Tran T. S. Fardeau J. C. Giroux M. (1988). Effects of soil properties on plant-available phosphorus determined by the isotopic dilution phosphorus-32 method. Soil Sci. Soc. America J. 52, 1383–1390. doi: 10.1007/s11368-010-0329-9

[B30] Van Soest P. J. Robertson J. B. Lewis B. A. (1991). Methods for dietary fiber, neutral detergent fiber, and nonstarch polysaccharides in relation to animal nutrition. J. dairy Sci. 74, 3583–3597. doi: 10.3168/jds.S0022-0302(91)78551-2 1660498

[B31] Venter Z. S. Jacobs K. Hawkins H.-J. (2016). The impact of crop rotation on soil microbial diversity: A meta-analysis. Pedobiologia 59, 215–223. doi: 10.1016/j.pedobi.2016.04.001

[B32] Wang Y. C. Dang N. Feng K. Wang J. B. Jin X. Yao S. T.. (2023). Grass-microbial inter-domain ecological networks associated with alpine grassland productivity. Front. Microbiol. 14. doi: 10.3389/fmicb.2023.1109128 PMC9905801 36760496

[B33] Weidhuner A. Zandvakili O. R. Krausz R. Crittenden S. J. Deng M. Hunter D.. (2022). Continuous no-till decreased soil nitrous oxide emissions during corn seasons after 48 and 50 seasons in a poorly-drained Alfisol. Sci. Total Environ. 838, 156296. doi: 10.1016/j.scitotenv.2022.156296 35660440

[B34] Xian Y. Cai G. Lin J. Chen Y. Wang X. (2023). Comparison of crop productivity, economic benefit and environmental footprints among diversified multi-cropping systems in South China. Sci. Total Environ. 874, 162407. doi: 10.1016/j.scitotenv.2023.162407 36858234

[B35] Xu L. Tang G. Liu J. Tian J. Wang X. Zhang J. (2021). Cover crops can produce livestock forage in Chinese subtropical regions. Agron. J. 113, 1535–1547. doi: 10.1002/agj2.20619

[B36] Yang L. Liao W. H. Gao Z. L. Wang S. Zhang J. R. Zhao Y. J. (2018). Production potentials of biomass and crude protein, and ammonia volatilizations under different forage crop rotations. J. Soil ang Water Conserv. 32, 312–320. doi: 10.13870/j.cnki.stbcxb.2018.02.046

[B37] Ye J. H. Wang Y. H. Lin S. X. Wang Y. C. Chen P. Y. Hong L.. (2023). Metabolomics analysis of the effect of acidification on rhizosphere soil microecosystem of tea tree. Front. Plant Sci. 14. doi: 10.3389/fpls.2023.1137465 PMC9998672 36909384

[B38] Zhang J. Iwaasa A. D. Han G. Gu C. Wang H. Jefferson P. G.. (2018). Utilizing a multi-index decision analysis method to overall assess forage yield and quality of C3 grasses in the western Canadian prairies. Field Crops Res. 222, 12–25. doi: 10.1016/j.fcr.2018.03.007

[B39] Zhang X. Zhou Z. (2019). Quantifying effects of root systems of planted and natural vegetation on rill detachment and erodibility of a loessial soil. Soil Tillage Res. 195, 104420. doi: 10.1016/j.still.2019.104420

[B40] Zook D. M. Erwin D. C. Stolzy L. H. (1986). Anatomical, morphological, and physiological responses of alfalfa to flooding. Plant Soil 96, 293–296. doi: 10.1007/BF02374773

