# Supplementary material for: Yield and nutrient composition of forage crops and their effects on soil characteristics of winter fallow paddy in South China
Source: Front Plant Sci. 2024 Jan 16;14:1292114. doi: 10.3389/fpls.2023.1292114 (PMC10825004; doi:10.3389/fpls.2023.1292114)
Supplement: Supplementary file 1 [file Table_1.docx]

Table S1 Model construction for significant correlation (*P*<0.05, n=60) among crops and soil indicators

| Items | DMY | CPY | DM | CP | EE | CF | NDF | ADF | WSC | ADL | pH | AP | OM | TN | Ur | Ca | Acid–p | In | Bacteria | Actinomyces | Fungi | Azotobacter |
| --- | --- | --- | --- | --- | --- | --- | --- | --- | --- | --- | --- | --- | --- | --- | --- | --- | --- | --- | --- | --- | --- | --- |
| DMY | 1.000 |  |  |  |  |  |  |  |  |  |  |  |  |  |  |  |  |  |  |  |  |  |
| CPY | 0.778^**^ | 1.000 |  |  |  |  |  |  |  |  |  |  |  |  |  |  |  |  |  |  |  |  |
| DM | 0.247 | 0.146 | 1.000 |  |  |  |  |  |  |  |  |  |  |  |  |  |  |  |  |  |  |  |
| CP | -0.793^**^ | -0.324 | -0.2003 | 1.000 |  |  |  |  |  |  |  |  |  |  |  |  |  |  |  |  |  |  |
| EE | -0.236 | -0.242 | 0.090 | 0.356 | 1.000 |  |  |  |  |  |  |  |  |  |  |  |  |  |  |  |  |  |
| CF | -0.468^**^ | -0.604^**^ | -0.024 | 0.042 | -0.178 | 1.000 |  |  |  |  |  |  |  |  |  |  |  |  |  |  |  |  |
| NDF | 0.240 | 0.199 | -0.082 | -0.415^*^ | -0.704^**^ | 0.141 | 1.000 |  |  |  |  |  |  |  |  |  |  |  |  |  |  |  |
| ADF | 0.458^*^ | 0.491^**^ | 0.039 | -0.459^*^ | -0.796^**^ | 0.047 | 0.828^**^ | 1.000 |  |  |  |  |  |  |  |  |  |  |  |  |  |  |
| WSC | 0.647^**^ | 0.371^*^ | 0.301 | -0.449^*0^ | 0.381^*^ | -0.517^**^ | -0.353 | -0.254 | 1.000 |  |  |  |  |  |  |  |  |  |  |  |  |  |
| ADL | -0.734^**^ | -0.356 | -0.384^*^ | 0.656^**^ | -0.176 | 0.284 | 0.229 | 0.060 | -0.846^**^ | 1.000 |  |  |  |  |  |  |  |  |  |  |  |  |
| pH | -0.282 | -0.438^*^ | -0.261 | -0.176 | -0.440^*^ | 0.592^**^ | 0.531^**^ | 0.324 | -0.462^*^ | 0.446^*^ | 1.000 |  |  |  |  |  |  |  |  |  |  |  |
| AP | 0.011 | 0.361 | 0.185 | 0.379^*^ | 0.212 | -0.521^**^ | -0.539^**^ | -0.325 | 0.234 | -0.072 | -0.652^**^ | 1.000 |  |  |  |  |  |  |  |  |  |  |
| OM | 0.324 | 0.021 | -0.358 | -0.542^**^ | -0.509^**^ | 0.088 | 0.626^**^ | 0.453^*^ | -0.055 | -0.030 | 0.457^*^ | -0.682^**^ | 1.000 |  |  |  |  |  |  |  |  |  |
| TN | 0.024 | 0.322 | -0.052 | 0.161 | -0.245 | -0.283 | -0.117 | 0.063 | -0.164 | 0.049 | -0.464^**^ | 0.626^**^ | -0.209 | 1.000 |  |  |  |  |  |  |  |  |
| Ur | 0.305 | 0.472^**^ | 0.279 | 0.178 | 0.392^*^ | -0.681^**^ | -0.563^**^ | -0.300 | 0.607^**^ | -0.459^*^ | -0.886^**^ | 0.746^**^ | -0.483^**^ | 0.421^*^ | 1.000 |  |  |  |  |  |  |  |
| Ca | -0.066 | 0.323 | 0.159 | 0.408^*^ | 0.176 | -0.414^*^ | -0.440^*^ | -0.273 | 0.094 | -0.053 | -0.655^**^ | 0.885^**^ | -0.629^**^ | 0.730^**^ | 0.704^**^ | 1.000 |  |  |  |  |  |  |
| Acid–p | -0.760^**^ | -0.309 | -0.170 | 0.798^**^ | 0.094 | 0.162 | -0.252 | -0.279 | -0.622^**^ | 0.687^**^ | -0.033 | 0.418^*^ | -0.479^**^ | 0.422^*^ | 0.048 | 0.544^**^ | 1.000 |  |  |  |  |  |
| In | -0.044 | 0.436^*^ | -0.114 | 0.469^**^ | 0.049 | -0.439^*^ | -0.324 | -0.1206 | -0.013 | 0.125 | -0.605^**^ | 0.806^**^ | -0.473^**^ | 0.700^**^ | 0.643^**^ | 0.846^**^ | 0.530^**^ | 1.000 |  |  |  |  |
| Bacteria | 0.276 | -0.137 | -0.266 | -0.532^**^ | -0.142 | 0.130 | 0.176 | 0.141 | 0.131 | -0.231 | 0.350 | -0.528^**^ | 0.574^**^ | -0.325 | -0.413^*^ | -0.673^**^ | -0.608^**^ | -0.573^**^ | 1.000 |  |  |  |
| Actinomyces | 0.466^**^ | 0.301 | 0.562^**^ | -0.277 | 0.049 | -0.312 | -0.105 | 0.030 | 0.509^**^ | -0.531^**^ | -0.492^**^ | 0.263 | -0.054 | 0.160 | 0.477^**^ | 0.179 | -0.345 | -0.036 | 0.049 | 1.000 |  |  |
| Fungi | 0.478^**^ | 0.343 | 0.152 | -0.245 | 0.070 | -0.420^*^ | -0.137 | 0.032 | 0.516^**^ | -0.600^**^ | -0.504^**^ | 0.175 | 0.042 | 0.024 | 0.451^*^ | 0.123 | -0.337 | 0.108 | 0.150 | 0.583^**^ | 1.000 |  |
| Azotobacter | -0.525^**^ | -0.033 | -0.003 | 0.738^**^ | 0.022 | -0.048 | -0.193 | -0.195 | -0.392^*^ | 0.544^**^ | -0.204 | 0.591^**^ | -0.449^*^ | 0.458^*^ | 0.2701 | 0.634^**^ | 0.780^**^ | 0.610^**^ | -0.568^**^ | -0.056 | -0.2009 | 1.000 |

Note: Asterisks indicate significant differences at P < 0.05 (*) and P < 0.01 (**), respectively. DMY, dry matter yield; CPY, crude protein yield; DM, dry matter; CP, crude protein; EE, ether extract; CF, crude fiber; NDF, neutral detergent fiber; ADF, acid detergent fiber; WSC, water-soluble carbohydrates; ADL, acid detergent lignin; AP, available phosphorus; OM, organic matter; TN, total nitrogen; Ur, urease; Ca, catalase; Acid-p, acid-phosphatase; In, invertase.
